# DNA methylation levels of *RELN* promoter region in ultra-high risk, first episode and chronic schizophrenia cohorts of schizophrenia

**DOI:** 10.1038/s41537-022-00278-0

**Published:** 2022-10-10

**Authors:** Sok-Hong Kho, Jie Yin Yee, Shu Juan Puang, Luke Han, Christine Chiang, Attilio Rapisarda, Wilson Wen Bin Goh, Jimmy Lee, Judy Chia Ghee Sng

**Affiliations:** 1 Department of Pharmacology, Yong Loo Lin School of Medicine, National University of Singapore, Singapore, Singapore; 2 Lee Kong Chian School of Medicine, Nanyang Technological University, Singapore, Singapore; 3 Research Division, Institute of Mental Health, Singapore, Singapore; 4 Yong Loo Lin School of Medicine, National University of Singapore (NUS), Singapore, Singapore; 5 Neuroscience and Behavioural Disorders, Duke-NUS Medical School, Singapore, Singapore; 6 School of Biological Sciences, Nanyang Technological University, Singapore, Singapore; 7 Department of Psychosis, Institute of Mental Health, Singapore, Singapore

**Keywords:** Schizophrenia, Biomarkers

## Abstract

The essential role of the Reelin gene (*RELN*) during brain development makes it a prominent candidate in human epigenetic studies of Schizophrenia. Previous literature has reported differing levels of DNA methylation (DNAm) in patients with psychosis. Therefore, this study aimed to (1) examine and compare *RELN* DNAm levels in subjects at different stages of psychosis cross-sectionally, (2) analyse the effect of antipsychotics (AP) on DNAm, and (3) evaluate the effectiveness and applicability of *RELN* promoter DNAm as a possible biological-based marker for symptom severity in psychosis.. The study cohort consisted of 56 healthy controls, 87 ultra-high risk (UHR) individuals, 26 first-episode (FE) psychosis individuals and 30 chronic schizophrenia (CS) individuals. The Positive and Negative Syndrome Scale (PANSS) was used to assess Schizophrenia severity. After pyrosequencing selected CpG sites of peripheral blood, the Average mean DNAm levels were compared amongst the 4 subgroups. Our results showed differing levels of DNAm, with UHR having the lowest (7.72 ± 0.19) while the CS had the highest levels (HC: 8.78 ± 0.35; FE: 7.75 ± 0.37; CS: 8.82 ± 0.48). Significantly higher Average mean DNAm levels were found in CS subjects on AP (9.12 ± 0.61) compared to UHR without medication (UHR(−)) (7.39 ± 0.18). A significant association was also observed between the Average mean DNAm of FE and PANSS Negative symptom factor (*R*^**2**^ = 0.237, ß = −0.401, *p = 0.033). In conclusion, our findings suggested different levels of DNAm for subjects at different stages of psychosis. Those subjects that took AP have different DNAm levels. There were significant associations between FE DNAm and Negative PANSS scores. With more future experiments and on larger cohorts, there may be potential use of DNAm of the *RELN* gene as one of the genes for the biological-based marker for symptom severity in psychosis.

## Introduction

Schizophrenia affects more than 1% of the global population and is studied to be a complex multifactorial disease with a recognised genetic and environmental component^1^. However, the understanding of its aetiology remains incomplete. As its diagnosis generally depends on interview-based subjective assessments of self-reported symptoms, there is an urgent need to identify biomarkers to address the diagnosis and prognosis of schizophrenia^2^. Identifying biomarkers for psychosis will facilitate early diagnoses, interventions and personalised treatment strategies or regimes for individuals^3^.

In humans, genetic studies have reported that the Reelin (*RELN*) locus is associated with neuropsychiatric disorders like Schizophrenia, bipolar disorder and autistic spectrum disorder^4–6^. Reelin is a glycoprotein mainly secreted by cells and a subpopulation of GABAergic interneurons. It has been shown to play an essential role in the development of cortical neural connectivity at embryonic stages and synaptic plasticity at postnatal stages^7,8^. Reduced *RELN* mRNA and protein levels were found in brain and blood of patients with Schizophrenia^8^. Post-mortem studies have observed that the *RELN* mRNA and protein levels are reduced by ~50% in nearly every region of the cerebral cortex^9^, leading investigators to hypothesise that reduced *RELN* levels increase susceptibility to Schizophrenia. Additionally, Fatemi found that multiple psychotropic medications affected the mRNA and protein products of *RELN*, affecting the Reelin signalling system^10^. These changes were hypothesised to explain the efficacy of these medications in the treatment of Schizophrenia and supported the investigation of the *RELN* signalling system as therapeutic targets in the treatment of neuropsychiatric diseases.

There have been numerous studies on the involvement of epigenetic mechanisms in the transcriptional regulation of *RELN*^11^. One study reported hypermethylation (increased DNA methylation level) of *RELN* promoter regions in brain samples of individuals with Schizophrenia^12^. Another study by Chen et al. also observed that hypermethylation of *RELN* in specific CpG sites silenced mRNA expression of *RELN*^13^. However, there is a lack of such observation in other stages of psychosis, namely prodromal and first-episode psychosis. This investigation into *RELN* DNAm in various phases of psychosis will allow an understanding of *RELN* dysregulation and its involvement in the development of psychosis.

**Fig. 1:**
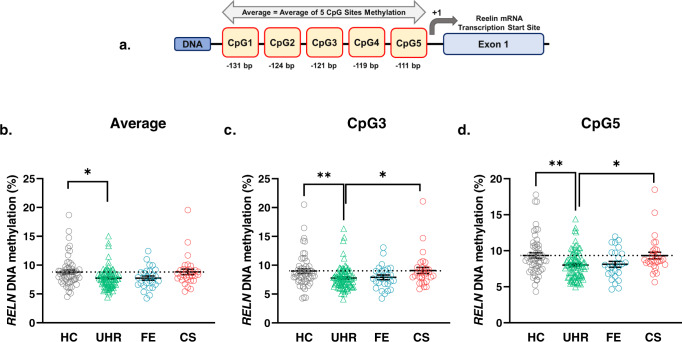
*RELN* DNA methylation levels among groups. **a** The human *RELN* gene exon I promoter schematic diagram and the five CpG sites in our study. The CpG sites’ locations were denoted by the base pair upstream of the Transcription Start Site of the *RELN* mRNA at +1. CpG1 (−131bp), CpG2 (−124), CpG3 (−121), CpG4 (−119), CpG5 (−111). (drawing not in scale). **b** Average mean DNAm of five CpG sites profiles of HC, UHR, FE and CS; **c** Mean DNAm of CpG3 of HC, UHR, FE and CS; **d** Mean DNAm of CpG5 of HC, UHR, FE and CS. Lines and whiskers in graphs represent mean ± standard error. Statistical test: Kruskal–Wallis non-parametric test for non-normal distribution of data sets followed by Dunn’s multiple comparison post hoc test. *p ≤ 0.05; **p ≤ 0.01. DNAm DNA methylation. HC healthy control. UHR ultra-high risk. FE first episode. CS chronic Schizophrenia. For detailed information on mean ± standard error and *p* values, see Supplementary S Table 2.

Therefore, the present study aims were to (1) Examine and compare *RELN* DNA methylation (DNAm) levels in candidate CpG sites (Fig. 1a) in subjects at different stages of psychosis: ultra high-risk (UHR), first episode (FE) and chronic schizophrenia (CS) cross-sectionally, (2) Analyse the effect of antipsychotic (AP) drugs on DNA methylation level (DNAm), (3) Evaluate the applicability of *RELN* promoter DNAm as a possible biomarker for symptom severity in psychosis.

## Materials and methods

### Study sample

Between 2005 and 2018, a total of 199 participants were recruited at the Institute of Mental Health (IMH), Singapore. Our study included four groups: (1) healthy control (HC), (2) individuals at ultra-high risk (UHR), (3) patients with first-episode psychosis (FE), and (4) patients with chronic Schizophrenia (CS). Ethics approval for this study was provided by the National Healthcare Group Domain Specific Review Board. All participants had the mental capacity to give informed consent. Guardian consents were obtained for participants under the age of 18.

HC were individuals with no known psychiatric (assessed by the Structured Clinical Interview for DSM-IV Axis I disorder) or neurological disorders. UHR comprised individuals who met the UHR criteria defined by the Comprehensive Assessment of At-Risk Mental State (CAARMS) score at the time of recruitment^14^. UHR was not on antipsychotic exposure of more than 5 mg haloperidol per day for 3 weeks (or equivalent) or were not on antipsychotics at the point of recruitment. FE included individuals who are recovering from their first psychotic episode and have had less than 4 weeks of antipsychotic treatment. CS consisted of individuals who fulfilled DSM-IV diagnosis of Schizophrenia with a treatment duration of more than 5 years.

The Positive and Negative Syndrome Scale (PANSS) was used to quantitate symptom severity in UHR, FE and CS groups by trained raters with established inter-rater reliability at >0.8. Socio-demographic data such as age, gender, ethnicity, body mass index (BMI), and use of medication were collected. Subjects that have incomplete PANSS scores or no DNA methylation records were removed from the analysis.

### DNA methylation and analysis—bisulfite conversion and pyrosequencing

A sample of whole blood was collected from all participants. Genomic DNA was extracted using a QIAamp^®^ DNA Blood Midi Kit (Qiagen, Hilden, Germany).

#### Bisulfite conversion

Bisulfite conversion was performed using an EpiTect^®^ Bisulfite Conversion Kit according to the manufacturer’s protocol (Qiagen). The bisulfite converted DNA was then amplified using primers [forward: 5′- GTTAGGGGTTTTAAGAAGGTGTGGA-3′ and reverse: 5′-ACTCCCAAAATTACTTTAAACC-3′ (biotinylated)] designed using Pyromark Assay design software version 2.0. Amplification was performed by QIAGEN PyroMark^®^ PCR Kit according to the manufacturer’s protocol.

#### Pyrosequencing

Amplified products underwent pyrosequencing, performed on PyroMark^®^ Q48 Autoprep Pyrosequencing system (Qiagen), using pyrosequencing primer [5′-GGGGAGGGAGTTTTTTTAGG-3′] (Integrated Data Technologies), and PyroMark^®^ Q48 Advanced CpG Reagents (Qiagen) and PyroMark^®^ Q48 Magnetic Beads (Qiagen) based on manufacturer’s protocol.

#### Analysis

DNA methylation (DNAm) (%) was obtained for each participant at identified CpG sites. In brief, the current study targeted five CpG sites within the promoter region of the *RELN* gene (−111 bp to −131 bp) (Fig. 1a). In this study, we defined “Average mean DNAm” as the average of all CpG sites 1 to 5. This form of reporting was adopted from previous methylome studies^15,16^. DNAm may be regulated differently due to differences in protein binding or factors at different CpG sites, which may lead to varying DNAm levels between CpG sites^17,18^. Thus, we also reported “mean DNAm” as the DNAm values of a single site.

### Statistical analyses

Graphical illustrations and statistical analysis were performed using GraphPad PRISM^®^ Version 9.3.1. (GraphPad Software, Inc., San Diego, CA, USA). Data were also analysed on SPSS Statistics version 23 (IBM Co., Armonk, NY, USA). Statistical significances were set at *p ≤ 0.05; **p ≤ 0.01; ***p ≤ 0.001; ****p ≤ 0.0001. Kruskal–Wallis test followed by Dunn’s multiple comparison post hoc test were used to compare mean DNAm between and were performed among four subject groups. A multivariate linear regression model was employed to examine the association between DNAm vs. PANSS and DNAm vs. duration of antipsychotic treatment (DOT).

## Results

### Participant demographics

Table 1 shows the demographics of the 4 subgroups, medications given and the PANSS scores of the cohorts. The 4 subgroups were the healthy control (HC), the ultra-high risk (UHR), the first-episode (FE) and chronic Schizophrenia (CS).

**Table 1:** Demographic table.

Group	*n*	Age (mean ± SEM)	Gender *n* and %		Ethnicity *n* and %				Medications	PANSS			
										Positive	Negative	General	Total
			Male	Female	Chinese	Indian	Malay	Others		(mean ± SEM)			
HC	56	36.57 ± 1.22	31	25	49	1	6	0	No	N.A.	N.A.	N.A.	N.A.
			55%	45%	88%	2%	11%	0%					
UHR	87	21.52 ± 0.38	31	56	62	6	16	3	(−) (38), AD (49)	10.92 ± 0.29	12.20 ± 0.42	25.94 ± 0.76	49.06 ± 1.21
			36%	64%	71%	7%	18%	3%					
FE	26	36.54 ± 1.48	14	12	23	1	2	0	AP (18), AP + AD (8)	10.88 ± 0.81	10.54 ± 0.79	23.42 ± 1.47	44.85 ± 2.77
			52%	46%	89%	4%	8%	0%					
CS	30	43.87 ± 1.35	17	13	28	0	2	0	AP (23), AP + AD (7)	9.93 ± 0.74	8.90 ± 0.52	18.80 ± 0.53	37.63 ± 1.41
			57%	43%	93%	0%	7%	0%					

PANSS (Total) = PANSS (Positive) + PANSS (Negative) + PANSS (General).

*HC* healthy control, *UHR* ultra-high risk, *FE* first episode, *CS* chronic Schizophrenia, *No Medication* (−), *AP* antipsychotic, *AD* antidepressant, *N.A.* Not Available

A total of 199 participants (93 males,106 females), which consisted of 4 subgroups: HC (56), UHR (87), FE (26) and CS (30) were recruited for this study. The participants had an almost equal number of males and females except for UHR, which comprised a higher number of females. The subjects were made up of 84.1% of Chinese, which accurately reflected the ethnic makeup of Singapore’s population. These 4 groups will be compared cross-sectionally.

For symptom severity, UHR was observed to have the highest PANSS scores when compared to FE and CS. UHR had more severe symptoms than CS with statistically significant higher positive (10.92 ± 0.29 vs. 9.93 ± 0.74, *p = 0.0453), negative (12.2 ± 0.42 vs. 8.9 ± 0.52, ****p < 0.0001), general (25.94 ± 0.76 vs. 18.8 ± 0.53, *p = 0.0234) and total PANSS scores than CS (49.06 ± 1.21 vs. 37.63 ± 1.41, ****p < 0.0001).

One of our aims was to compare the DNAm cross-sectionally across the 4 groups. We examined first whether BMI, gender and age affect the DNAm of these four groups of subjects.

The average BMI of the 4 groups were about the same: HC (23.06 ± 0.54), UHR (22.57 ± 0.56), FE (22.63 ± 0.78) and CS (25.52 ± 1.28). We checked the association of BMI with DNAm by linear regression and found that BMI had no association with DNAm (result not shown). For gender, we had about an equal number of males and females in each cohort except UHR, which has more females (see Table 1). We did ANOVA test, and we noted the only significant difference at CpG4 and corrected the result accordingly (data not shown). We did not study the effect of smoking as the data were incomplete. Regarding age, the result can be seen in S Table 5 and S Fig. 1. We separated the age of subjects in HC into less than 30 group, 31 to 40 group, 41 to 50 group, 51 to 60 group and 61 to 72 group. We then compared the DNAm between these groups. Generally, the DNAm decreased or became more hypomethylated with increasing age; however, the amount of hypomethylation was not significant in HC, albeit the wide range of age. In this study, we separated the patients into 3 groups according to different stages of Schizophrenia (e.g. UHR was younger than CS); this stratified the age; thus, we did not make adjustments for the age when compared cross-sectionally between these 3 groups.

### *RELN* DNA methylation levels among groups

The schematic diagram in Fig. 1a shows the five CpG sites we studied. These sites are between the transcription start site (TSS) and the enhancer region. This region is vital in binding factors that facilitate the interaction of long-range transcription factors at the enhancer and the polymerase and transcription machinery near the TSS^17,18^.

When we looked at the DNAm among the 4 groups, UHR was observed to have the lowest DNAm across all studied CpG sites (Fig. 1b, c, d and S Table 2). Below are notable findings across group comparisons.

#### Comparison between HC and UHR

UHR is noted to hypomethylated in all studied CpG sites when compared to HC but only differences were significant for sites, CpG 3 (8.99 ± 0.38 vs. 7.79 ± 0.21, **p = 0.0075), CpG5 (9.34 ± 0.36 vs. 8.01 ± 0.20, **p = 0.0058) and Average mean DNAm of all sites (8.78 ± 0.35 vs. 7.72 ± 0.19, *p = 0.0214) only (Fig. 1b, c, d and S Table 2).

#### Comparison between HC and FE

FE was observed to be consistently hypomethylated in all studied CpG sites compared to HC, but this difference is not statistically significant.

#### Comparison between HC and CS

We observed that CS DNAm levels were less hypomethylated and were almost the same level as HC.

#### Comparison between UHR and CS

With UHR having the lowest DNAm levels in all sites, it has statistically significant lower levels than CS for CpG3 (7.79 ± 0.21 vs. 9.08 ± 0.53, *p = 0.0286) and CpG5 (8.01 ± 0.20 vs. 9.33 ± 0.46, *p = 0.0266) see Fig. 1c, d and S Table 2.

The trends we observed were interesting when considering the results of PANSS scores in Table 1. The PANSS scores from UHR were the highest (more severe) among FE and CS; however, UHR DNAm were the lowest of the three compared to HC. CS scored the lowest (less severe) of PANSS scores, but its DNAm were at the highest at about the same level as HC. This trend may indicate that DNAm and PANSS scores had some inverse correlation.

### *RELN* DNA methylation levels among groups according to the use of APs

We had observed a significant difference in DNAm levels between UHR and CS. Thus, we attempted to investigate if antipsychotic intake is the driving force behind these differences. We further filtered groups according to their medication regime: (1) Selecting only UHR not on psychotrophics [UHR(−)], (2) selecting FE on antipsychotics only (FE AP), and (3) selecting CS on antipsychotics only (CS AP).

We observed that UHR(−) had the lowest DNAm in all CpG sites among all subgroups. Below are notable differences among subgroups.

#### Comparison between HC vs. UHR(−)

**Fig. 2:**
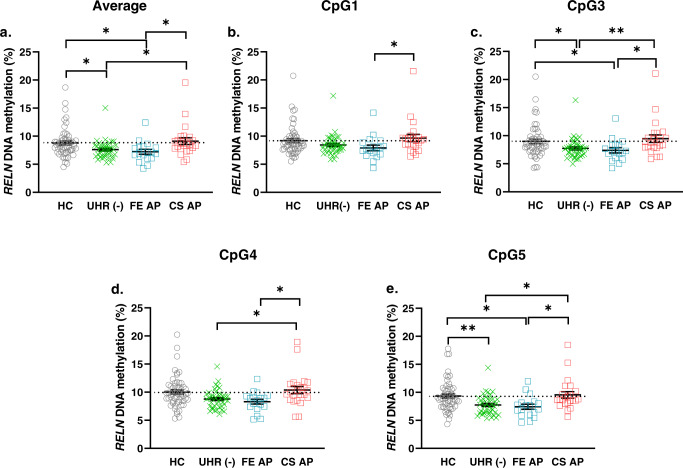
*RELN* DNA methylation levels among groups according to the use of APs. **a** Average mean DNAm of five CpG sites profiles of HC, UHR(−), FE AP and CS AP; **b** Mean DNAm of CpG1 of HC, UHR(−), FE AP and CS AP; **c** Mean DNAm of CpG3 of HC, UHR(−), FE AP and CS AP; **d** Mean DNAm of CpG4 of HC, UHR(−), FE AP and CS AP; **e** Mean DNAm of CpG5 of HC, UHR(−), FE AP and CS AP. Lines and whiskers in graphs represent mean ± standard error. Statistical test: Kruskal–Wallis non-parametric test for non-normal distribution of data sets followed by Dunn’s multiple comparison post hoc test. *p ≤ 0.05; **p ≤ 0.01. DNAm DNA methylation. HC healthy control. UHR(-), ultra-high risk with No medication. FE AP first-episode treated with antipsychotic. CS AP chronic Schizophrenia treated with antipsychotic. For detailed information on mean ± standard error and *p* values, see Supplementary S Table 3.

We found that UHR(−) were significantly hypomethylated when compared to HC at Average mean DNAm for all sites (8.78 ± 0.34 vs. 7.39 ± 0.18, *p = 0.017), CpG3 (8.99 ± 0.38 vs. 7.50 ± 0.20, *p = 0.0131), CpG5 sites (9.34 ± 0.36 vs. 7.55 ± 0.24, **p = 0.0021) (Fig. 2a, c, e and S Table 3).

#### Comparison between UHR(−) and CS AP

We found that UHR(−) were also significantly hypomethylated when compared to CS AP at CpG3 (7.50 ± 0.20 vs. 9.47 ± 0.67, **p = 0.0094), CpG4 (8.61 ± 0.24 vs. 10.38 ± 0.63, *p = 0.0238), CpG5 (7.55 ± 0.24 vs. 9.54 ± 0.58, *p = 0.0104) and Average mean DNAm of all sites (7.39 ± 0.18 vs. 9.12 ± 0.61, *p = 0.0181) (Fig. 2a, c, d, e and S Table 3).

#### Comparison between FE AP and CS AP

We also observed that DNAm levels of FE AP were significantly lower when compared to CS AP in all CpG sites and Average mean DNAm of all sites (CpG1: 7.91 ± 0.50 vs. 9.67 ± 0.64, *p = 0.0355, CpG3: 7.39 ± 0.47 vs. 9.47 ± 0.67, *p = 0.0211, CpG4: 8.30 ± 0.43 vs. 10.38 ± 0.63, *p = 0.0345, CpG5: 7.42 ± 0.45 vs. 9.54 ± 0.58, *p = 0.0198 and Average mean DNAm of all sites: 7.22 ± 0.43 vs. 9.12 ± 0.61, *p = 0.0201) (Fig. 2a, b, c, d, e and S Table 3).

These differences in DNAm levels may be due to the treatment of antipsychotics (AP). To find out whether the treatment effects of AP affect the mean DNAm levels of these CpG sites, we correlated the duration of antipsychotic treatment (DOT) with the mean DNAm. Not much significance was found for both cohorts FE AP and CS AP, (S Table 6a, b, respectively). However, there were interesting trends: For FE AP, the subjects were at the early stage of treatment (less than 4 weeks of APs treatment), and not much correlation with DNAm was found (S Table 6a). In CS AP, the DOT seemed to have stronger correlation with DNAm, as seen in CpG5; (Spearman’s rank, *r* = 0.357, *p* = 0.103) (S Table 6b). CS AP slightly stronger correlation to DNAm could be because CS AP cohorts took AP for a much longer period than FE AP.

### PANSS scores among groups with APs

Recall the DNAm of UHR, FE and CS seemed to have an inverse relationship with the PANSS scores. Next, we checked the PANSS scores of these three groups with APs. We wanted to know if the PANSS scores of the cohorts showed the same inverse trend with the DNAm.

**Fig. 3:**
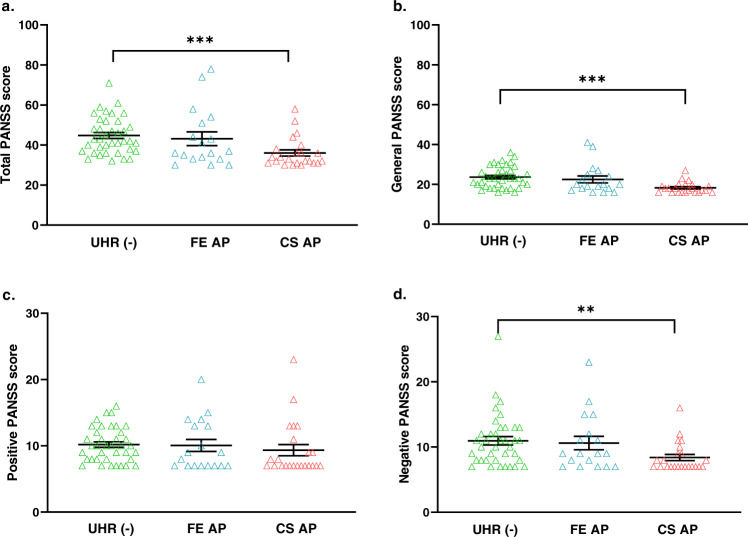
PANSS scores among groups with APs. **a** Total PANSS scores of UHR(−), FE AP and CS AP; **b** General PANSS scores of UHR(−), FE AP and CS AP; **c** Positive PANSS scores of UHR(−), FE AP and CS AP; **d** Negative PANSS scores of UHR(−), FE AP and CS AP. Lines and whiskers in graphs represent mean ± standard error. Statistical test: Kruskal–Wallis non-parametric test for non-normal distribution of data sets followed by Dunn’s multiple comparison post hoc test.**p ≤ 0.01; ***p ≤ 0.001. HC healthy control. UHR(−) ultra-high risk with No medication. FE AP first-episode treated with antipsychotic. CS AP chronic Schizophrenia treated with antipsychotic. For detailed information on mean ± standard error and *p* values, see Supplementary S Table 4.

In Fig. 3, we observed that the UHR(−) had the highest PANSS scores in Total, General, Positive and Negative, and CS AP had the lowest. The PANSS scores for UHR(-) and CS AP were significantly different in Total (44.76 ± 1.49 vs. 36.04 ± 1.55, ***p = 0.0002), General (23.63 ± 0.88 vs. 18.30 ± 0.58, ***p = 0.0001) and Negative PANSS scores (10.95 ± 0.65 vs. 8.39 ± 0.47, **p = 0.0040) (see S Table 4). FE AP scores the second highest in all 4 categories. This trend was inversely related to the DNAm profiles of the UHR(-), FE AP and CS AP in Fig. 2.

It seemed that antipsychotic treatments might lower scores in two symptoms of Schizophrenia, the negative and general symptoms. However, the patients also received other non-medication therapies like counselling, etc., so their PANSS scores were the total efforts of these treatments. DNA methylations, too, were affected by medications and other environmental factors. To know how well the DNAm is associated with the PANSS scores, we did a correlation of DNAm and PANSS scores and then linear regression.

### Association of *RELN* DNA methylation with PANNS scores

To explore the association between *RELN* DNAm and PANSS, we employed multivariate linear regressions (controlling for age and gender) between DNAm levels and PANSS for respective groups. If the association was strong, the association indicated that DNAm could associate with higher biological functions like behaviours.

We observed significant associations between *RELN* DNAm at CpG2 (*R*^2^ = 0.227, ß = −0.388, *p = 0.039), CpG3 (*R*^2^ = 0.233, ß = −0.398, *p = 0.034), CpG5 (*R*^2^ = 0.306, ß = −0.408, *p = 0.024) and Average mean DNAm of all sites (*R*^2^ = 0.237, ß = −0.401, *p = 0.033) with PANSS negative score (Table 2).

**Table 2:** Tabulated results of the association of *RELN* DNA methylation with PANSS scores.

Relationship	After adjustment for age and gender				
Association	Adjusted *R*^2^	Beta	*p*	Lower 95% CI	Higher 95% CI
CpG2 FE DNAm vs. P(−)	0.227	−0.388	0.039	−0.308	−0.009
CpG3 FE DNAm vs. P(−)	0.233	−0.398	0.034	−0.392	−0.017
CpG5 FE DNAm vs. P(−)	0.306	−0.408	0.024	−0.388	−0.031
Ave FE DNAm vs. P(−)	0.237	−0.401	0.033	−0.358	−0.017
CpG5 FE AP DNAm vs. P(−)	0.23	−0.468	0.057^a^	−0.419	0.007

Adjusted R square, beta, *p* of Linear Regression and upper and lower 95% Confidence Interval (CI); after adjustment for age and gender. Association of DNAm with PANSS scores.

*FE* first episode, *P (−)* Negative PANSS scores.

^a^Denotes near significant.

The *R*^**2**^ suggested that for around 30.6% of the population of FE, their DNAm at CpG5 could explain or predict the Negative PANSS score. Beta values describe the prediction’s strength direction, and a higher value reflects a stronger prediction. A negative beta value of −0.408 means that there was an inverse relation. That meant methylation increased by 1 unit; the Negative PANSS score decreased by 0.408 units. Similar deductions could be drawn from the association of CpG2, CpG3 and Average of five sites. As shown in Fig. 1, as the mean DNAm values increased from UHR to FE, Negative PANSS scores in Table 1 decreased from UHR to FE, which showed an inverse relationship. These results from the association (see Table 2) indicated that FE DNAm could predict Negative behaviours (PANSS score).

As for DNAm with PANSS scores for the cohorts who took AP, we associated FE AP with PANSS scores and adjusted for age and gender. FE AP at CpG5 had a close to significant association with Negative PANSS scores (see Table 2); adjusted *R*^**2**^ = 0.23, Beta = −0.468, *p* = 0.057.

In summary, for our data set, FE DNAm data associated well with the Negative behaviours of the FE patients. Likewise, FE AP DNAm also showed some association with FE AP Negative PANSS scores, and the result was close to significant.

## Discussion

Our finding indicated that there were hypomethylations of CpG sites in *RELN* promoter for patients with Schizophrenia when compared to healthy control. UHR and FE showed more hypomethylation at the CpG sites, while some CS individuals showed hypomethylation, and some CS DNAm were almost the same level as HC.

However, studies focused on the *RELN* gene differed from our findings, as they showed significant hypermethylation amongst patients with Schizophrenia. One study by Abdolmaleky using post-mortem brain samples found that CpG islands at the *RELN* promoter showed significantly higher levels of methylation in patients with Schizophrenia compared to controls^12^. Another study by Nabil, using peripheral blood samples, reported a significantly higher level of *RELN* promoter in patients with Schizophrenia as compared to healthy controls^19^. In both these studies, the expression of the *RELN* gene was found to be silenced by the high levels of DNAm in patiens with Schizophrenia. The different *RELN* CpG sites used may explain this discordance between our study and that observed by Abdolmaleky and Nabil. In this study, we analysed five *RELN* promoter CpG sites from −111 bp to −131 bp between the enhancer region and the transcription start site. In contrast, the studies by Abdolmaleky and Nabil analysed different CpG sites at *RELN* promoter. These different CpG sites highlight another observation we made during these experiments that every CpG site was slightly different. We should report them separately rather than just the Average mean DNAm of the five CpG sites reported in most methylome data^15,16^ (see Fig. 1b compared to Fig. 1c, d).

One report by Lintas analysed DNAm levels at the same sites as our CpG1, CpG2, CpG4 and CpG5 sites. However, this study only focused on the DNAm from post-mortem samples of the neocortex of healthy individuals—which yielded similar results of DNAm levels to those in our HC subgroup^20^. Thus, it seemed like our results were consistent with Lintas.

Our findings of DNA hypomethylation in patients with Schizophrenia were consistent with studies that analysed other genes that affect Schizophrenia. Murata reported that a significantly lower average global DNA methylation level of the CpG sites of the LINE-1 promoter was observed in first-episode Schizophrenia patients compared to controls in the well-cohort. In addition, this study found that DNAm levels were inversely correlated with scores on the global assessment of functioning (GAF) scale (*R* = −0.543, *p = 0.011 in Spearman’s rank correlation)^21^. This correlation study echoed our inversed correlation of DNAm with the PANSS scores in this study.

In another finding, a study found that hypomethylation of the CpG site cg19647197 within the CCDC53 gene was associated with patients with Schizophrenia who were suicide attempters compared to the Schizophrenia suicide non-attempters^22^. Another study by Alfimova found a positive association between DNA hypomethylation and cognitive index, suggesting the role of hypomethylation in the development of cognitive deficits in Schizophrenia^23^.

Another concern of our results was regarding age’s effect on the changes in DNAm levels. CpG sites DNAm can be hypermethylated or hypomethylated. And as a person ages, CpG sites can be increasingly hypo or hypermethylated. Attributing the increase in DNAm from FE levels to CS levels to age would assume that the five CpG sites became hypermethylated with age. In our study, the five CpG sites were hypomethylated as age increased, shown in the HC group (S Table 5 and S Fig. 1), covering a wide range of ages. This hypomethylation at the five CpG sites with age reinforced that the increase in DNAm could be attributed to treatment, not ageing.

One minor observation we had for our results was the question of accelerated ageing in Schizophrenia. Studies have found that DNAm changes as part of natural aging and can be measured via DNAm clocks to observe the effect of diseases like cancer and Down’s syndrome^24,25^. Schizophrenia is proposed to accelerate epigenetic aging^26^, which is linked to psychosis severity^27^. Our results showed that the five CpG sites in HC are hypomethylated with increasing age (S Table 5 and S Fig. 1). For UHR cohort, they were even more hypomethylated at the same age range (data not shown), agreeing with accelerated DNA methylation ageing in disease models^26–28^. However, a study by Mckinney has found otherwise, leaving the effect of Schizophrenia on DNAm age uncertain^28^.

Our results also showed the possible effects of antipsychotic treatment on both mean DNAm levels and PANSS scores. Antipsychotics have been found to be epigenetic modifiers, on a site-specific and genome-wide level^29^. Our findings demonstrated a correlation between antipsychotics in increasing the DNAm levels and reducing symptom severity. Our results are consistent with a study by Melas, which compares peripheral blood leucocyte samples of healthy controls and participants with Schizophrenia. The study showed a significant association between antipsychotic treatment and higher global DNAm levels in patients with Schizophrenia. Antipsychotic treated samples more closely resemble the DNAm levels of healthy control participants^30^. In other DNA sites such as Interleukin-6 (IL-6), the hypomethylated state of the IL-6 promoter of patients with Schizophrenia were shown to be reversed by treatment with antipsychotics^31^. Though our findings agreed with the results of other researchers, there were some limitations. Firstly, the AP taken by the patients were different. Some APs are known to increase DNAm while some decrease DNAm^32,33^. Secondly, most patients were taking other medications together with the APs, which might affect the DNAm levels^34^. This possibly explains our result; why the DOT of AP did not correlate well with CS AP DNAm, even with CS AP taking AP for a more extended period.

There was disagreement between our findings and Dong on the effects of antipsychotics on the methylation of mice reelin promoters. They indicated decreased levels of DNAm at the *Reln* promoters for the mice treated with clozapine but not haloperidol^33^. However, this study used the promoter regions of mouse *Reln* and was conducted with normal mouse brain tissue that had been induced to mimic the psychosis phenotype in mice. In contrast, our study utilised peripheral blood samples in healthy controls and patients with Schizophrenia.

Regarding the type of samples used, a literature review showed that peripheral blood reflects the same methylation trends of post-mortem brain samples. A study by Grayson using post-mortem samples from the prefrontal cortex showed hypermethylation at positions −139 (CpApG) and −134 (CpTpG) within the *RELN* promoter in patients with Schizophreia compared to their controls^11^. A similar study by Nabil using peripheral blood samples showed similar hypermethylation results^19^. These findings suggest that DNAm in the peripheral blood may reflect the DNAm levels found in post-mortem brain samples. The use of peripheral blood samples is further reinforced by other epigenetic studies that propose that epigenetic changes can also be detected in peripheral tissues such as blood in psychiatric illnesses^30,35,36^. Although peripheral epigenetics may not be a perfect mirror image of the brain epigenetics ^37–39^, these findings, along with the scarcity of brain samples, provide a compelling basis for the use of peripheral blood samples as a DNAm marker for Schizophrenia.

The possible usage for our findings could be to identify the ultra-high risk state (UHR) for psychosis. UHR is a state where an individual might experience subclinical psychotic-like symptoms prior to the first episode of psychosis. Early intervention and preventive approaches such as the Scandinavian early treatment and intervention in psychosis study (TIPS) have shown significantly improved functional outcomes^40^. Identification of UHR individuals is performed via clinical interviews using validated measures such as the CAARMS^14^ or the Structured Interview for Prodromal Syndromes (SIPS)^41^. Although they boast a good overall prognostic performance, with a high sensitivity of 95%, these semi-structured interviews for psychosis prediction, like SIPS, have a low specificity of 47%^42,43^. Such UHR criteria may be unable to identify individuals with absent or few UHR symptoms.

The current limitations in clinical-based identification of UHR individuals highlight the potential role of biological-based markers to aid in the identification and subsequent commencement of the early intervention. Our results demonstrated that mean DNAm was significantly lower in the UHR or UHR(-) subgroups compared to HC. (S Table 2, S Table 3). Our result suggested the possible use of these five CpG sites of the *RELN* gene in identifying UHR individuals from their healthy counterparts in an asymptomatic population. Our study only covers a limited observation period of 2 years; only 9 out of 87 (10.3%) of the UHR subgroup converted to FE status. Of all the 9, 8/9 were female, and only 1/9 was male. Incidentally, these 9 individuals, as shown in S Fig. 2, 7/9 (77.8%) were from the UHR group that took AD and 2/9 (22.2%) from UHR(−) with no medications. Notice that those individuals in UHR AD primarily had DNAm around the mean DNAm value while those in UHR(-) had DNAm close to the lowest values. This data showed that DNAm might be sensitive to picking up UHR individuals.

Further longitudinal studies, longer than 2 years of observation period and with a larger group could examine the evolution, progression and conversion of UHR individuals to FE status. However, these five CpG sites of *RELN* were not only the CpG sites for identifying the UHR group. Maybe in the future, with other genes and other CpG sites, a panel of biological-based markers could be used in identifying the UHR cohort. Regarding the practicalities of using DNAm as a biological-based marker, it should be noted that the differences in DNAm levels are often minimal. The Average mean DNAm between the HC and UHR groups in this study were 8.78 ± 0.35 and 7.72 ± 0.19, respectively, a difference of only 1% (S Table 2). Therefore, we propose a standardised approach to reading the values of DNAm, where the raw value of DNAm is multiplied by 10. For example, HC DNAm levels of 87.8 compared to UHR DNAm levels of 77.2. This approach to reading DNAm values could potentially highlight the statistically significant differences in clinical practice.

The PANSS (Positive and Negative Syndrome Scale)^44^ is commonly used in clinical practice to score the severity of Schizophrenia. In a systematic review by Obermeier published in 2011, over 62% of the authors in published studies used incorrect calculations^45^. Thus, there is a need for a panel of biological-based markers to complement this existing clinical assessment method. Our findings showed a significant relationship between the negative PANSS score and DNAm levels in individuals with first-episode Schizophrenia. These results suggested that DNAm levels could provide a biological-based marker that correlates with the severity of schizophrenic symptoms, especially in the Negative symptoms. However, again, we should use more than one gene and more than a few CpG sites for this assessment.

There were a few limitations to our study. One limitation would be that the majority of our subjects are ethnically Chinese, limiting the generalisability of our results. However, this Chinese majority accurately reflects the ethnic makeup of Singapore’s population. The small cohort size and recruitment from a single centre may also be a limitation. However, the centre sees the largest pool of patients with Schizophrenia in Singapore, which also has a limited pool of appropriate participants within its relatively small population.

Lastly, there was no PANSS score data and no family history collected for the HC group. Obtaining the PANSS score and family history for the HC would have allowed us to assess the participants better and verify their clinical status as healthy controls.

## Conclusion

In conclusion, when we studied the DNAm of the five CpG sites of *RELN* promoter, we found differences between HC and cohorts of UHR, FE and CS. Patients who were administered with AP also showed different DNAm profiles. Moreover, the DNAm correlated well with Negative PANSS scores. The DNAm at these five CpG sites warrants further studies. It could be one of the CpG sites of a panel of genes used as the biological-based markers for identifying and assessing symptoms severity for patients with Schizophrenia.

## Supplementary information

Supplementary Figure Legends

S Table 1

S Table 2

S Table 3

S Table 4

S Table 5

S Table 6

S Figure 1

S Figure 2

## Data Availability

The data sets generated during and/or analysed during the current study are available from the corresponding author on reasonable request.

## Code availability

The R code we used for normalising and averaging triplicates of pyrosequencing results are available from the corresponding author on reasonable request.
