# Supplementary material for: DNA methylation levels of RELN promoter region in ultra-high risk, first episode and chronic schizophrenia cohorts of schizophrenia
Source: Schizophrenia (Heidelb). 2022 Oct 10;8(1):81. doi: 10.1038/s41537-022-00278-0 (PMC9550813; doi:10.1038/s41537-022-00278-0)
Supplement: Supplementary file 1 — Supplementary Figure Legends [file 41537_2022_278_MOESM1_ESM.pdf]

Supplementary figure legend

S Table 1

Medications treatment for different cohorts.

n = number of subjects. % = percentage of the cohort treated with the medication.

S Table 2

RELN DNA methylation values for Figure 1b, c, d. mean ± SEM = mean ± standard error. Statistical test: Kruskal-Wallis non-parametric test for non-normal distribution of data sets followed by Dunn's multiple comparison post hoc test. DNAm, DNA methylation. HC, healthy control. UHR, ultra-high risk. FE, first episode. CS, chronic Schizophrenia.

S Table 3

RELN DNA methylation values for Figure 2 a, b, c, d, e. mean ± SEM = mean ± standard error. Statistical test: Kruskal-Wallis non-parametric test for non-normal distribution of data sets followed by Dunn's multiple comparison post hoc test. DNAm, DNA methylation. HC, healthy control. UHR(-), ultra-high risk with No medication. FE AP, first episode treated with antipsychotic. CS AP, chronic Schizophrenia treated with antipsychotic.

S Table 4

PANSS scores for Figure 3 a, b, c, d. mean ± SEM = mean ± standard error. Statistical test: Kruskal-Wallis non-parametric test for non-normal distribution of data sets followed by Dunn's multiple comparison post hoc test. Total = Total PANSS scores. General = General PANSS scores. Positive = Positive PANSS scores. Negative = Negative PANSS scores. UHR(-), ultra-high risk with No medication. FE AP, first episode treated with antipsychotic. CS AP, chronic Schizophrenia treated with antipsychotic.

S Table 5

RELN DNA methylation of each CpG site across the age range of healthy control.

mean ± SEM = mean ± standard error. n = number of subjects. Statistical test: Kruskal-Wallis non-parametric test for non-normal distribution of data sets followed by Dunn's multiple comparison post hoc test. Found no significant difference.

S Table 6

Correlation of Duration of Antipsychotic Treatment of with *RELN* DNA methylation.

a. First Episode treated with antipsychotics (FE AP), Duration of antipsychotic treatment (DOT) correlated with mean DNAm of CpG1, CpG2, CpG3, CpG4, CpG5 and Average. b. Chronic Schizophrenia treated with antipsychotics (CS AP), Duration of antipsychotic treatment (DOT) correlated with mean DNAm of CpG1, CpG2, CpG3,

CpG4, CpG5 and Average. DOT, Duration of antipsychotic treatment in the unit of year; FE AP, first episode treated with antipsychotics; CS AP, chronic Schizophrenia treated with antipsychotics. Statistical test: Non-parametric Spearman's rank correlation for non-normal distribution of data sets, r = Spearman's rank correlation coefficient. 2 tailed p-value. CI, Confidence Interval. Found no significant association

S Figure 1

RELN DNA Hypomethylation of CpG1 to CpG5 with increasing age in healthy control

Mean DNA methylation of each CpG site across the different age ranges of healthy control. a. Average mean DNAm of five CpG sites across the different age ranges of HC; b. Mean DNAm of CpG1 across the different age ranges of HC; c. Mean DNAm of CpG2 across the different age ranges of HC; d. Mean DNAm of CpG3 across the different age ranges of HC; e. Mean DNAm of CpG4 across the different age ranges of HC; f. Mean DNAm of CpG5 across the different age ranges of HC. Lines in graphs represent mean ± standard error. Statistical test: Kruskal-Wallis non-parametric test for non-normal distribution of data sets followed by Dunn's multiple comparison post hoc test. DNAm, DNA methylation. HC, healthy control. For detailed information on mean ± standard error and p values, see Supplementary S Table 5. Found no significant association.

S Figure 2

RELN DNAm of converters among UHR subgroups of UHR with no medication [UHR(-)] and UHR who took antidepressants (UHR AD).

Converters are UHR individuals who had converted to the First Episode (FE) state.

a. Average mean DNAm of five CpG sites of UHR subgroups and HC (HC, mean ± standard error =8.78 ± 0.34; UHR(-), mean ± standard error =7.39 ± 0.18; HC vs UHR(-), $*p = 0.0173$). b. Mean DNAm of CpG3 of UHR subgroups and HC (HC, mean ± standard error = 8.99 ± 0.38; UHR(-), mean ± standard error =7.50 ± 0.20; HC vs UHR(-), $*p = 0.0131$). c. Mean DNAm of CpG5 of UHR subgroups and HC (HC, mean ± standard error = 9.34 ± 0.36; UHR(-), mean ± standard error =7.55 ± 0.24; HC vs UHR(-), $**p=0.0021$). DNAm HC as a comparison to the DNAm levels of the UHR subgroups. Lines and whiskers in graphs represent mean ± standard error. Statistical test: Kruskal-Wallis test followed by Dunn's multiple comparison post hoc test. $*p \leq 0.05$; $**p \leq 0.01$. DNAm, DNA methylation. HC, healthy control. UHR(-), ultra-high risk with No medication. UHR AD, ultra-high risk with antidepressants. The purple colours dots and squares denoted the converters in UHR(-) and UHR AD subgroups, respectively.
