# Supplementary material for: DNA methylation levels of RELN promoter region in ultra-high risk, first episode and chronic schizophrenia cohorts of schizophrenia
Source: Schizophrenia (Heidelb). 2022 Oct 10;8(1):81. doi: 10.1038/s41537-022-00278-0 (PMC9550813; doi:10.1038/s41537-022-00278-0)
Supplement: Supplementary file 2 — S Table 1 [file 41537_2022_278_MOESM2_ESM.pdf]

Medications \ Groups & number of subjects		Ultra-High Risk (UHR)<br>87	First Episode Psychosis (FE)<br>26	Chronic Schizophrenia (CS)<br>30
**Antipsychotics (AP)**	Aripiprazole			6.8% (2)
	Paliperidone			3.4% (1)
	Risperidone		50.0% (13)	13.8% (4)
	Clozapine		3.8%(1)	17.2% (5)
	Olanzapine		11.5%(3)	
	Sulpiride		7.7%(2)	13.8% (4)
	Modecate		3.8%(1)	
	Stelazine		3.8%(1)	10.3% (3)
	Clopixol			3.4% (1)
	chlorpromazine			3.4% (1)
	Piportil			3.4% (1)
	Haloperidol		3.8%(1)	
	Fluanxol		3.8%(1)	6.8%(2)
**Antidepressants (AD)**	Fluvoxamine	19.5% (17)		
	Fluoxetine	17.2% (15)		
	Escitalopram	6.9% (6)		
	Dothiepin	1.1% (1)		
	Mirtazapine	5.7% (5)		
	Amitriptyline	2.3% (2)		
**AP + AD**			Risperidone + Fluoxetine (7.7%) (2)<br>Risperidone + Fluvoxamine (3.8%) (1)<br>Risperidone + Lexapro (3.8%) (1)<br>Olanzapine + Escitalopram (3.8%) (1)<br>Sulpiride + Fluvoxamine (3.8%) (1)<br>Quetiapine + Fluoxetine (3.8%) (1)<br>Unknown (3.8%) (1)	Clozapine + Fluoxetine (6.8%) (2)<br>Aripiprazole + Fluoxetine (3.4%) (1)<br>Stelazine + Clomipramine (3.4%) (1)<br>Sulpiride + Dothiepin (3.4%) (1)<br>Fluanxol + Fluoxetine (3.4%) (1)<br>Haloperidol + Amitriptyline (3.4%) (1)

S Table 1
