# Supplementary material for: DNA methylation levels of RELN promoter region in ultra-high risk, first episode and chronic schizophrenia cohorts of schizophrenia
Source: Schizophrenia (Heidelb). 2022 Oct 10;8(1):81. doi: 10.1038/s41537-022-00278-0 (PMC9550813; doi:10.1038/s41537-022-00278-0)
Supplement: Supplementary file 3 — S Table 2 [file 41537_2022_278_MOESM3_ESM.pdf]

# Mean DNAm values for Figure 1 b,c,d

| | CpG1 (mean ± SEM) | CpG2 (mean ± SEM) | CpG3 (mean ± SEM) | CpG4 (mean ± SEM) | CpG5 (mean ± SEM) | Average (mean ± SEM) |
|---|---|---|---|---|---|---|
| **HC** | 9.20 ± 0.35 | 6.35 ± 0.34 | 8.99 ± 0.38 | 10.03 ± 0.37 | 9.34 ± 0.36 | 8.78 ± 0.35 |
| **UHR** | 8.50 ± 0.22 | 5.48 ± 0.17 | 7.79 ± 0.21 | 8.84 ± 0.19 | 8.01 ± 0.20 | 7.72 ± 0.19 |
| **FE** | 8.45 ± 0.40 | 5.43 ± 0.32 | 7.90 ± 0.41 | 8.80 ± 0.39 | 8.12 ± 0.41 | 7.75 ± 0.37 |
| **CS** | 9.43 ± 0.51 | 6.25 ± 0.47 | 9.08 ± 0.53 | 10.02 ± 0.51 | 9.33 ± 0.46 | 8.82 ± 0.48 |

| | CpG1 | CpG2 | CpG3 | CpG4 | CpG5 | Average |
|---|---|---|---|---|---|---|
| **HC vs UHR** | – | – | **$p = 0.0075$ | – | **$p = 0.0058$ | *$p = 0.0214$ |
| **UHR vs CS** | – | – | *$p = 0.0286$ | – | *$p = 0.0266$ | – |

**S Table 2**
