# Supplementary material for: DNA methylation levels of RELN promoter region in ultra-high risk, first episode and chronic schizophrenia cohorts of schizophrenia
Source: Schizophrenia (Heidelb). 2022 Oct 10;8(1):81. doi: 10.1038/s41537-022-00278-0 (PMC9550813; doi:10.1038/s41537-022-00278-0)
Supplement: Supplementary file 4 — S Table 3 [file 41537_2022_278_MOESM4_ESM.pdf]

Mean DNAm values for Figure 2 a,b,c,d,e.

	CpG1 (mean±SEM)	CpG2 (mean±SEM)	CpG3 (mean±SEM)	CpG4 (mean±SEM)	CpG5 (mean±SEM)	Average (mean±SEM)
HC	9.20±0.35	6.35±0.34	8.99±0.38	10.03±0.37	9.34±0.36	8.78±0.34
UHR(-)	8.16±0.19	5.15±0.17	7.50±0.20	8.61±0.24	7.55±0.24	7.39±0.18
FE AP	7.91±0.50	5.09±0.40	7.39±0.47	8.30±0.43	7.42±0.45	7.22±0.43
CS AP	9.67±0.64	6.54±0.60	9.47±0.67	10.38±0.63	9.54±0.58	9.12±0.61

	CpG1	CpG2	CpG3	CpG4	CpG5	Average
HC vs UHR(-)	–	–	*p = 0.0131	–	**p = 0.0021	*p = 0.0173
HC vs FE AP	–	–	*p = 0.0437	–	*p = 0.0105	*p = 0.0283
UHR (-) vs CS AP	–	–	**p = 0.0094	*p = 0.0238	*p = 0.0104	*p = 0.0181
FE AP vs CS AP	*p = 0.0355	–	*p = 0.0211	*p = 0.0345	*p = 0.0198	*p = 0.0201

S Table 3
