# Supplementary material for: DNA methylation levels of RELN promoter region in ultra-high risk, first episode and chronic schizophrenia cohorts of schizophrenia
Source: Schizophrenia (Heidelb). 2022 Oct 10;8(1):81. doi: 10.1038/s41537-022-00278-0 (PMC9550813; doi:10.1038/s41537-022-00278-0)
Supplement: Supplementary file 5 — S Table 4 [file 41537_2022_278_MOESM5_ESM.pdf]

PANSS scores for Figure 3 a, b, c , d

	Positive (mean±SEM)	Negative (mean±SEM)	General (mean±SEM)	Total (mean±SEM)
UHR(-)	10.18±0.42	10.95±0.65	23.63±0.88	44.76±1.49
FE AP	10.61±0.91	10.61±1.03	22.50±1.73	43.17±3.42
CS AP	9.35±0.85	8.39±0.47	18.30±0.58	36.04±1.55

	Positive	Negative	General	Total
UHR(-) vs CS AP	–	***p* = 0.0040	****p* = 0.0001	****p* = 0.0002

S Table 4
