# Supplementary material for: DNA methylation levels of RELN promoter region in ultra-high risk, first episode and chronic schizophrenia cohorts of schizophrenia
Source: Schizophrenia (Heidelb). 2022 Oct 10;8(1):81. doi: 10.1038/s41537-022-00278-0 (PMC9550813; doi:10.1038/s41537-022-00278-0)
Supplement: Supplementary file 6 — S Table 5 [file 41537_2022_278_MOESM6_ESM.pdf]

Mean DNAm of each CpG site across different Age Range of HC

	Age Range (years old)				
	<30 n=16	31-40 n=28	41-50 n=6	51-60 n=4	61-70 n=2
CpG1 (mean ± SEM)	9.84 ± 0.89	9.07 ± 0.40	7.97 ± 0.36	8.43 ± 1.16	11.08 ± 3.46
CpG2 (mean ± SEM)	6.64 ± 0.80	6.40 ± 0.46	5.31 ± 0.27	6.01 ± 0.91	7.28 ± 2.33
CpG3 (mean ± SEM)	9.28 ± 0.91	8.80 ± 0.45	8.04 ± 0.54	9.00 ± 1.53	12.03 ± 4.45
CpG4 (mean ± SEM)	9.91 ± 0.65	9.84 ± 0.54	10.03 ± 0.60	10.36 ± 1.85	13.10 ± 3.27
CpG5 (mean ± SEM)	9.33 ± 0.74	9.19 ± 0.49	8.56 ± 0.56	9.90 ± 1.54	12.76 ± 4.08
Average (mean ± SEM)	9.00 ± 0.78	8.66 ± 0.44	7.98 ± 0.43	8.74 ± 1.38	11.25 ± 3.52

S Table 5
