# Supplementary material for: DNA methylation levels of RELN promoter region in ultra-high risk, first episode and chronic schizophrenia cohorts of schizophrenia
Source: Schizophrenia (Heidelb). 2022 Oct 10;8(1):81. doi: 10.1038/s41537-022-00278-0 (PMC9550813; doi:10.1038/s41537-022-00278-0)
Supplement: Supplementary file 7 — S Table 6 [file 41537_2022_278_MOESM7_ESM.pdf]

Correlation of Duration of Antipsychotic Treatment (DOT) with *RELN* DNA methylation

a.

	FE AP DOT correlate with					
	CpG1	CpG2	CpG3	CpG4	CpG5	Ave
r	0.074	0.062	-0.104	-0.076	-0.004	0.016
p	0.784	0.818	0.7	0.78	0.989	0.954
95% CI	-0.4506 to 0.5608	-0.4600 to 0.5525	-0.5809 to 0.4264	-0.5618 to 0.4494	-0.5110 to 0.5044	-0.4955 to 0.5197

b.

	CS AP DOT correlate with					
	CpG1	CpG2	CpG3	CpG4	CpG5	Ave
r	0.117	0.242	0.286	0.315	0.357	0.257
p	0.604	0.278	0.196	0.153	0.103	0.249
95% CI	-0.3323 to 0.5230	-0.2129 to 0.6104	-0.1667 to 0.6397	-0.1357 to 0.6580	-0.08983 to 0.6836	-0.1979 to 0.6202

S Table 6
