# Supplementary figures and images for: DNA methylation levels of RELN promoter region in ultra-high risk, first episode and chronic schizophrenia cohorts of schizophrenia
Source: Schizophrenia (Heidelb). 2022 Oct 10;8(1):81. doi: 10.1038/s41537-022-00278-0 (PMC9550813; doi:10.1038/s41537-022-00278-0)

# Hypomethylation of CpG1 to CpG5 with Increasing Age

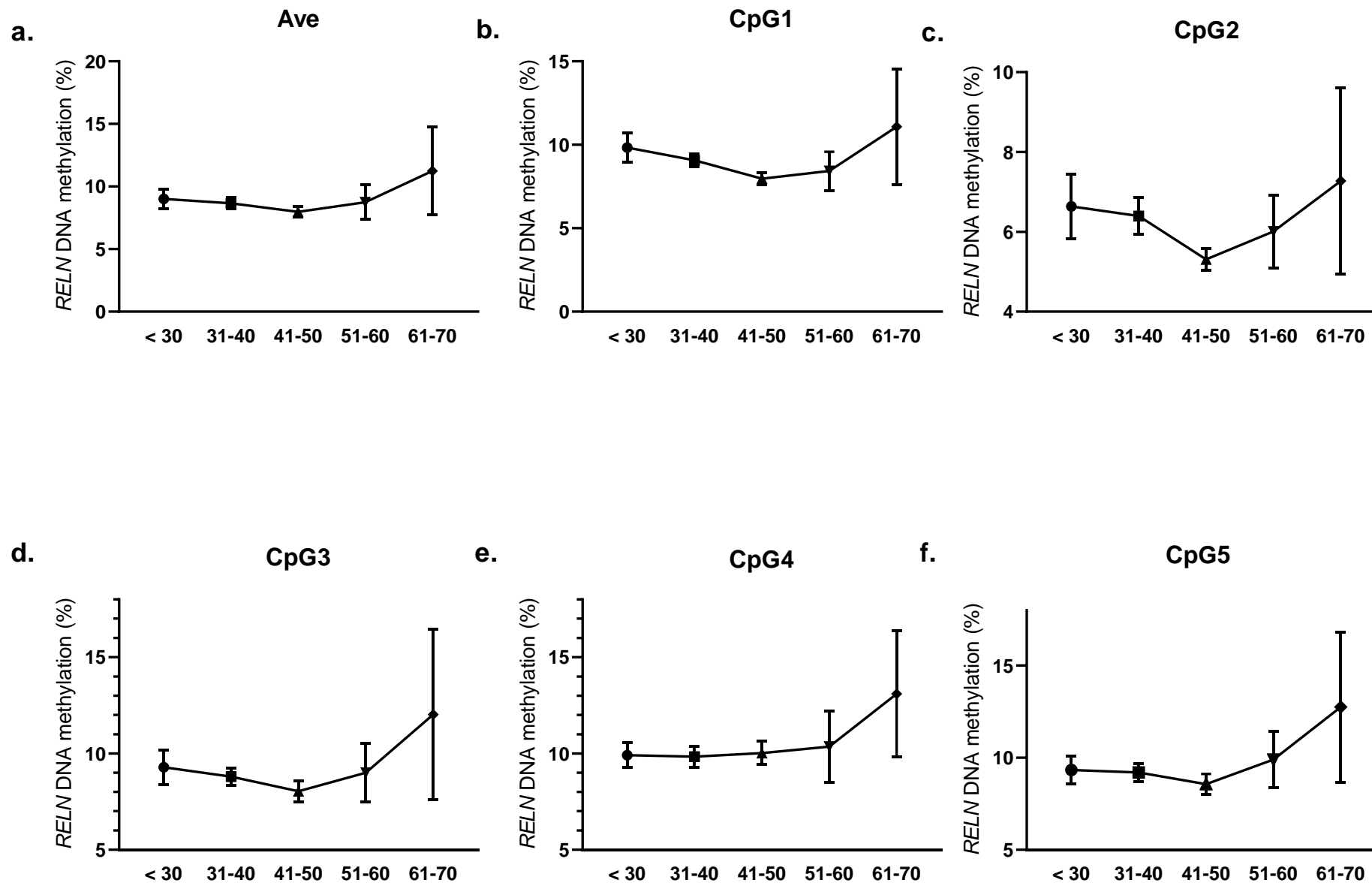

Supplement: Supplementary file 8 — S Figure 1 [file 41537_2022_278_MOESM8_ESM.pdf]

## *RELN* DNAm of converters among UHR subgroups

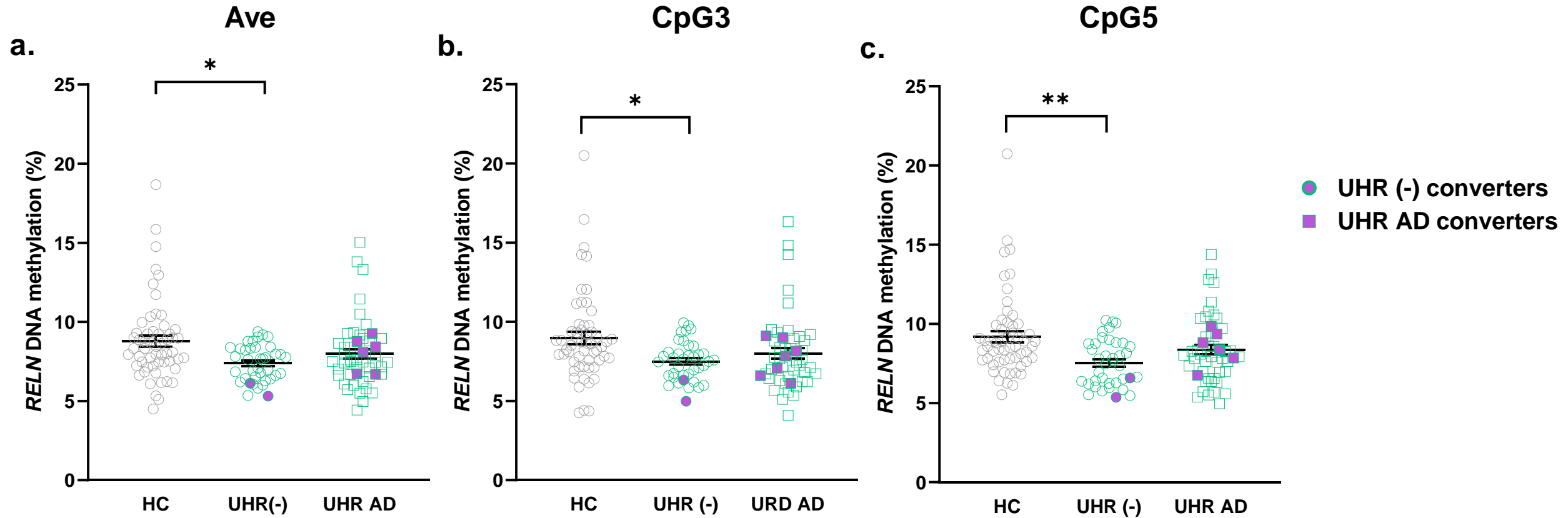

Supplement: Supplementary file 9 — S Figure 2 [file 41537_2022_278_MOESM9_ESM.pdf]
